# Acute Spontaneous Subdural Hematoma in a Patient With an End-Stage Renal Disease After Starting Dual Antiplatelet Therapy Post Drug-Eluting Stent Insertion: A Case Report

**DOI:** 10.7759/cureus.41761

**Published:** 2023-07-12

**Authors:** Aliaa Mousa, Ahmed Hassan, Bashar Oudah, Kudret Isilay Arslan, Pirouz Parang

**Affiliations:** 1 Internal Medicine, Capital Health Regional Medical Center, Trenton, USA; 2 Internal Medicine, Eisenhower Medical Center, Rancho Mirage, USA; 3 Cardiology, Capital Health Regional Medical Center, Trenton, USA

**Keywords:** pci, chest pain, liver transplant, peritoneal dialysis (pd), aspirin and ticagrelor (dapt), acute spontaneous subdural hematoma

## Abstract

Dual antiplatelet therapy (DAPT) has been widely utilized for secondary prevention in patients with cardiovascular diseases, such as post-drug eluting stent insertion, stroke, and peripheral vascular disease. The occurrence of bleeding complications, including intracranial hemorrhage, has been extensively studied in relation to DAPT. However, the occurrence of acute spontaneous subdural hematomas in this context is relatively rare. These hematomas can manifest through various symptoms, including altered mental status (AMS) and confusion. The risk of intracranial hemorrhage is particularly higher in patients receiving aspirin with ticagrelor, especially in those with reduced estimated glomerular filtration rate (eGFR) and liver disease.

In this case report, we present the case of a patient with end-stage renal disease undergoing peritoneal hemodialysis and a remote history of liver transplant. The patient presented to the hospital with chest pain, subsequently underwent drug-eluting stent placement, and was initiated on DAPT. Following the initiation of DAPT, the patient developed confusion and was diagnosed with an acute spontaneous subdural hematoma. The patient underwent middle meningeal artery embolization to manage the hematoma.

## Introduction

The administration of dual antiplatelet therapy (DAPT), combining acetylsalicylic acid (ASA) and an oral P2Y12 inhibitor, has become standard practice following coronary artery stenting, playing a crucial role in secondary prevention for patients with acute coronary syndrome (ACS) [1,2]. This therapeutic approach has significantly reduced the incidence of re-infarction and stent thrombosis after percutaneous coronary intervention (PCI). However, a major concern associated with antiplatelet therapy is the increased risk of internal bleeding. Among the various bleeding complications, intracranial hemorrhage poses a particular threat to patients receiving antiplatelet therapy following PCI. Acute spontaneous subdural hematoma (ASSDH), characterized by the absence of trauma, is a rare but critical condition that necessitates timely recognition and appropriate management.

## Case presentation

**Figure 1:**
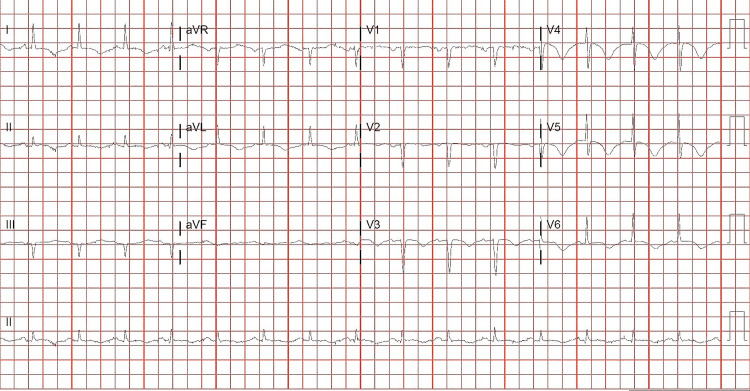
Electrocardiogram showing sinus rhythm with left atrial enlargement, old inferior infarction and anterior infarction, prolonged QT interval 577.

A 64-year-old male with a past medical history of hypertension, liver transplant secondary to hepatitis C, and hepatocellular carcinoma for which he is on tacrolimus, end-stage renal disease (ESRD) on peritoneal dialysis, cardiac tamponade status post pericardial window in a previous admission, presented to the emergency department overnight with shortness of breath, nausea, and dizziness for the past three weeks before presentation. The patient at that time denied any cough, sore throat, congestion, chest pain, or fever. He was complaining of word-finding difficulty (Anomia) on rare occasions for a couple of weeks before the presentation. On the initial physical exam, he had normal heart sounds, bilateral crackles at lower lung bases, peritoneal dialysis catheter in place with no surrounding erythema, and lower extremity edema +1. Neuro exam showed alert, awake, and oriented x4 (AAO x4), no abnormal findings of cranial nerves II-XII, and normal speech. No motor deficits are noted, with muscle strength 5/5 bilaterally. The sensation is intact bilaterally. Electrocardiogram (EKG) (Figure 1) showed sinus rhythm with left atrial enlargement, old inferior infarction and anterior infarction, and prolonged QT interval 577.

**Figure 2:**
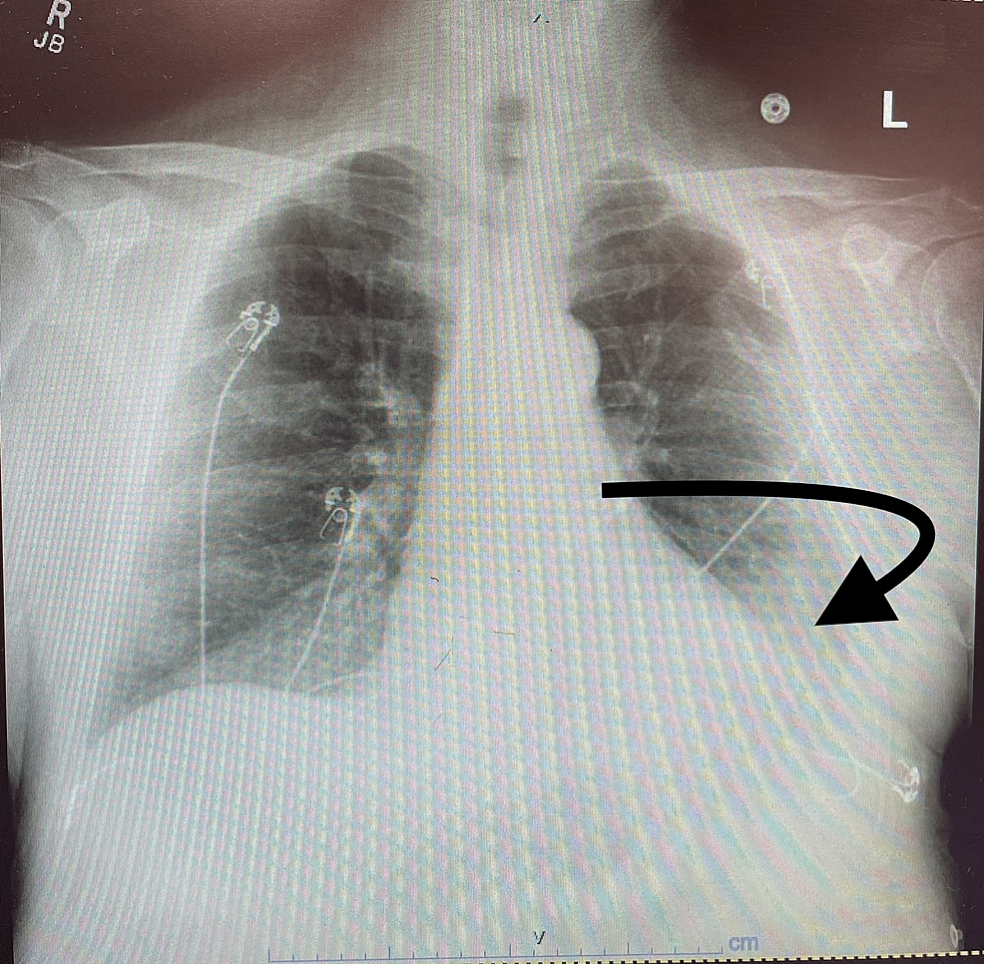
Chest x-ray showing mild to moderate left pleural effusion, and atelectasis in the left lung base.

Chest x-ray (Figure 2) showed mild to moderate left pleural effusion, and atelectasis in the left lung base. Initial troponin was 1.69 and has risen to 2.328, and remained flat, brain natriuretic peptide (BNP) was over 160,000. Vital signs on presentation were blood pressure 132/86 mmHg, temperature 36.9 degrees, heart rate 95 bpm, and respiratory rate 20/min, the patient was on room air saturating at 100%.

**Figure 3:**
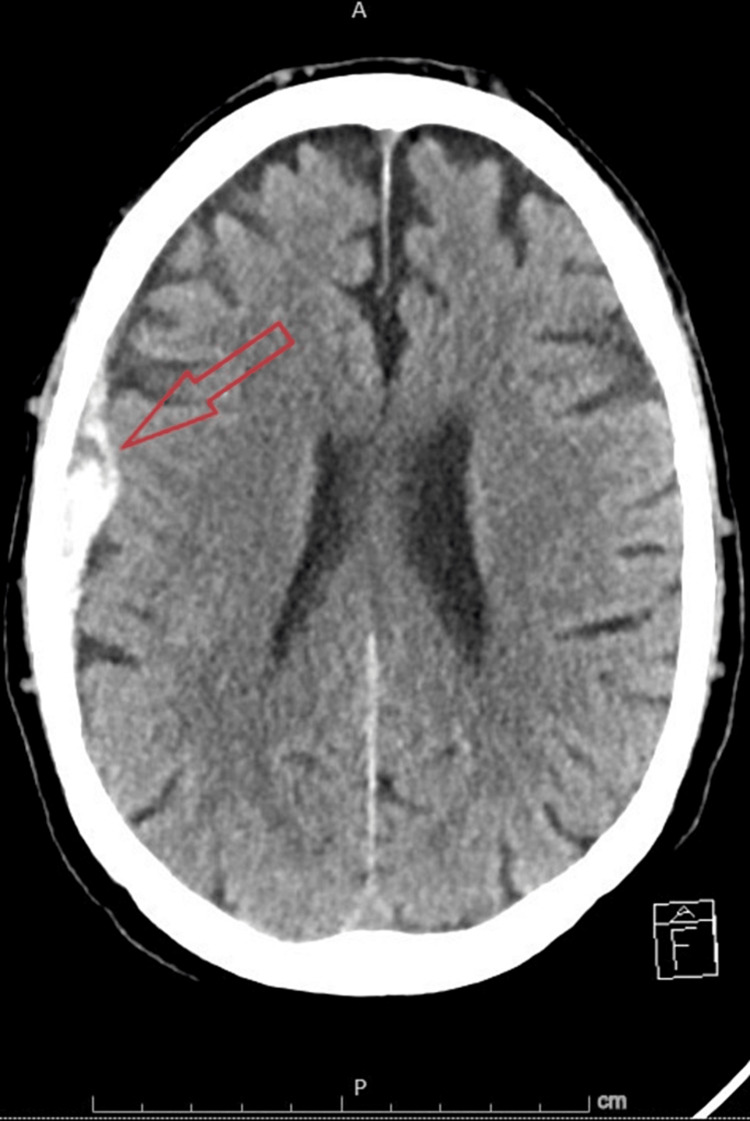
CT of the head showing acute right convexity subdural hematoma measuring up to 11 mm in maximal diameter with midline shift to the left measuring up to 2 mm.

At that time, the patient had a loading dose of aspirin 325 mg, and 4,000 units of heparin, and was started on a heparin drip per ACS protocol for non-ST-elevation myocardial infarction (STEMI), also one dose of intravenous Lasix 40 mg was given. At that time, the patient was also started on daily aspirin 81 mg, atorvastatin 40 mg daily at bedtime, carvedilol 12.5 mg twice daily, transthoracic echocardiography (TTE) was requested. TTE showed normal left ventricular size with severe systolic dysfunction. There is severe hypokinesis to akinesis of the distal 1/2 of the left ventricle, worse in the anterior and anteroseptal walls. EF 25%-30%, moderate tricuspid regurgitation, trivial pericardial effusion, compared with a previous echo that was done a month earlier. He had a computed tomography (CT) of the head without contrast that showed no acute intracranial abnormalities, but neurology service was consulted for the complaint of word-finding difficulty, by that time and after 67544444444444complete assessment and reviewing of the whole work up, anomia was not appreciated. On the same day, the initial diagnosis by cardiology was Takotsubo cardiomyopathy secondary to consistent wall motion abnormalities on TTE and left heart catheterization (LHC) was requested and also heparin was stopped earlier. Before LHC, he got a loading dose of Ticagrelor 180 mg, and LHC showed early-mid and distal left anterior descending artery (LAD) disease with drug-eluting stent placement x1 each. Later that day (after four hours from the loading dose), the patient started to develop cognitive impairment and deteriorated from being AAO x4 on presentation to AAO x1, oriented to himself only, and requiring one-to-one observation at the bedside. That was attributed to hyperactive delirium. Also, hepatic encephalopathy was excluded due to normal ammonia and bilirubin level. We received recommendations for frequent orientation and sleep cycle preservation and as needed melatonin during the night and to avoid benzodiazepines. The following night, the patient remained combative and had a repeat CT (Figure 3) head that showed an acute right convexity subdural hematoma (SDH) measuring up to 11 mm in maximal diameter with a trace midline shift to the left measuring up to 2 mm, which is new compared with the most recent prior head CT. Immediately aspirin, Ticagrelor, and prophylaxis subcutaneous heparin were stopped, and the patient was upgraded to the neuro intensive care unit where his neuro exam was significant for being lethargic, oriented only to himself, but was not clear with location or date, prosodic without dysarthria, the face appeared symmetric and strength was 4/5 throughout. Neurosurgery service was consulted and recommended to repeat CT head in the morning which showed no interval changes from the one overnight. On the following day, he was transferred to a tertiary care center for embolization of the middle meningeal artery. After that procedure, he was transferred back to our institute, being fully oriented, with no gross neurological deficit. At that time DAPT has been on hold for five days, so he got a loading dose of both aspirin 325 mg and Plavix 300 mg, cardiology recommended to continue with DAPT therapy for a one-year post-discharge with a very close follow-up. Furthermore, the patient was discharged home after being evaluated and optimized by both physical and occupational therapy.

## Discussion

DAPT is widely recognized as a fundamental treatment approach for various patients with cardiovascular disease. Including individuals undergoing PCI, those experiencing ACS, individuals with high-risk chronic coronary artery disease [1,2], and patients with a recent history of stroke or transient ischemic attack (TIA) [3]. The use of DAPT, specifically the combination of clopidogrel and aspirin, has been associated with an increased risk of acute SDH [4]. Several studies have explored this association and have provided valuable insights into the incidence and risk factors associated with DAPT-induced SDH. The risk of SDH associated with DAPT, including both clopidogrel plus aspirin and ticagrelor plus aspirin, highlights the importance of carefully weighing the benefits and risks of antiplatelet therapy in individual patients [5]. Factors such as age, sex, body weight, renal function, baseline anemia, and previous history of intracerebral hemorrhage (ICH) have been identified as potential risk factors for major bleeding with antiplatelet therapy [6].

One study conducted by Connolly et al. [7] analyzed data from randomized clinical trials and reported that the incidence of SDH in patients on aspirin therapy was 0.02/1,000 patient-years. The odds ratio for SDH in this group was 1.6, suggesting a slightly increased risk compared to those not on aspirin. Wong et al. [8] further noted that the location of lobar hematoma was higher in the aspirin group (32.8%) compared to the control group (10.3%). Bakheet et al. [9] performed a meta-analysis of 11 randomized clinical trials investigating the risk of SDH in patients receiving DAPT (clopidogrel plus aspirin). Although eight trials did not show any cases of spontaneous SDH, three trials with 23,136 participants reported 39 cases of SDH during a mean follow-up of 2.1 years per patient. The authors concluded that while the absolute rate of SDH in patients on DAPT was relatively low (1.1/1,000 patient-years), there was a higher risk of major bleeding with clopidogrel plus aspirin compared to aspirin plus placebo or aspirin alone.

The introduction of ticagrelor, a newer reversible P2Y12 receptor antagonist, has also been associated with an increased risk of bleeding, including intracranial bleeding and SDH. In the Platelet Inhibition and Patient Outcomes (PLATO) [10] trial, ticagrelor was compared to clopidogrel in patients with ACS. The trial reported similar rates of major bleeding between the two groups but noted a higher incidence of intracranial hemorrhage in the ticagrelor arm (0.3% versus 0.2%). Furthermore, fatalities due to bleeding were more frequent in the ticagrelor group (0.12% versus 0.0%). While ticagrelor demonstrated a net clinical benefit in terms of cardiovascular outcomes, the increased risk of bleeding, including SDH, should be considered.

It is noteworthy that patients treated with ticagrelor, particularly those with a low baseline estimated glomerular filtration rate (eGFR) below 30 mL/min, had a higher likelihood of experiencing a major bleeding event (19.0%) compared to patients with eGFR ≥30 mL/min (10.3%) [5]. In contrast, the increase in the risk of major bleeding for clopidogrel patients with low eGFR was not as significant (11.3% versus 9.9%, respectively). Furthermore, patients with eGFR below 30 mL/min had a higher risk of major bleeds with ticagrelor compared to clopidogrel (23 (19%) versus 16 (11.3%), number needed to harm (NNH) = 12, 95% confidence interval (CI): -6-514). What is more concerning is that ticagrelor-treated patients with eGFR below 30 mL/min were more likely to experience death (31 (26.5%) versus 34 (23.4%), NNH = 35, 95% CI: -7-13) and develop renal failure (12 (13.6%) versus 5 (5.4%), NNH = 15, 95% CI: 7-156) compared to clopidogrel [11]. Therefore, until further evidence is provided, ticagrelor should not be administered to patients with eGFR below 30 mL/min. Additionally, patients with mild liver disease at baseline had a 28% increased risk of major bleeds with ticagrelor compared to clopidogrel (11.2% versus 8.8%, relative risk = 1.28, 95% CI: 0.72-2.30) [12]. Thus, caution should be exercised when prescribing ticagrelor to individuals with mild liver disease. It is important to note that the PLATO trial excluded patients with moderate to severe liver disease, and therefore, individuals with this level of liver disease should not receive ticagrelor.

It is crucial to note that while the incidence of SDH and bleeding events associated with antiplatelet therapy is relatively low on an individual level, the cumulative burden of these events on a population level can be significant due to the widespread use of DAPT in conditions such as ACS and stroke. The lack of a rapid reversal agent for antiplatelet agents further complicates the management of bleeding events and contributes to the morbidity and mortality associated with SDH in these patients [13]. Bentracimab, a high-affinity monoclonal antibody fragment, has demonstrated promising results in reversing the antiplatelet effect of ticagrelor and its active metabolite [14]. In phase I trial, intravenous administration of bentracimab had a dramatic reversal of ticagrelor's antiplatelet effect within minutes, lasting up to 20 hours. These findings prompted the FDA to grant bentracimab breakthrough therapy designation, expediting its review and approval process for the treatment of serious or life-threatening conditions.

The REVERSE-IT trial [15] aims to evaluate the effectiveness of bentracimab in ticagrelor-treated patients and undergoing urgent surgery or invasive procedures, or with severe bleeding that cannot be controlled. Successful outcomes from this trial could potentially eliminate the need to discontinue ticagrelor for extended periods (more than five days) when patients undergo high-risk invasive interventions. Regarding the resumption of antiplatelet therapy after ICH, the optimal timing remains unclear.

The question regarding the safety of resuming antiplatelet therapy after intracerebral bleeding was investigated in the RESTART trial [16]. In this study, patients who had experienced intracerebral bleeding while on antithrombotic therapy were randomly assigned to either resume or discontinue antiplatelet agents. On average, patients who resumed single antiplatelet therapy did so approximately 2.5 months after the initial intracerebral bleed. The trial results showed no significant difference in the rates of recurrent symptomatic spontaneous ICH between the two groups. Interestingly, there was a trend indicating potentially lower bleeding rates among patients who resumed antiplatelet therapy.

In the same trial [16], only a small proportion of patients (3%) resumed DAPT (aspirin + clopidogrel) after ICH. In the absence of data guiding providers, the use of DAPT in patients following ICH should generally be avoided unless specific circumstances warrant it. For instance, patients who underwent PCI within one month of the ICH may require DAPT to minimize the risk of stent thrombosis.

## Conclusions

The use of DAPT, particularly ticagrelor plus aspirin, has been associated with an increased risk of ASSDH. The incidence of SDH is relatively low but can have significant implications on both individual patients and the population as a whole. Clinicians should carefully assess the benefits and risks of antiplatelet. And coming up with new reversal agents should be deeply looked into. 
